# A Novel Neotropical *Bacillus siamensis* Strain Inhibits Soil-Borne Plant Pathogens and Promotes Soybean Growth

**DOI:** 10.3390/microorganisms13061366

**Published:** 2025-06-12

**Authors:** Rodrigo F. Moreira, Elizabeth B. E. Pires, Odaiza F. Sousa, Giselly B. Alves, Luis O. Viteri Jumbo, Gil R. Santos, Luís J. Maia, Bergmann M. Ribeiro, Guy Smagghe, Elvio H. B. Perino, Rudolf Hausmann, Eugenio E. Oliveira, Raimundo W. S. Aguiar

**Affiliations:** 1 Programa de Pós-graduação em Biotecnologia, Universidade Federal do Tocantins (UFT), Gurupi 77402-970, TO, Brazilelizabethpires11.22@gmail.com (E.B.E.P.); luis.viteri@mail.uft.edu.br (L.O.V.J.); guysma9@gmail.com (G.S.); 2 Departamento de Biotecnologia, Universidade Federal do Tocantins, Gurupi 77410-530, TO, Brazil; 3 Programa de Pós-graduação em Produção Vegetal, Universidade Federal de Tocantins (UFT), Gurupi 77402-970, TO, Brazil; 4 Departamento de Biologia Celular, Instituto de Biologia, Universidade de Brasília (UnB), Brasília 70910-900, DF, Brazil; 5 Institute of Entomology, Guizhou University, Guiyang 550025, China; 6 Department of Plants and Crops, Ghent University, 9000 Ghent, Belgium; 7 Department of Biology, Vrije Universiteit Brussel (VUB), 1050 Brussels, Belgium; 8 Department of Bioprocess Engineering, Institute of Food Science and Biotechnology, University of Hohenheim, Fruwirthstr. 12, 70599 Stuttgart, Germany; 9 Departamento de Entomologia, Universidade Federal de Viçosa, Viçosa 36570-900, MG, Brazil

**Keywords:** biocontrol, fungal pathogens, soybean, sustainability, sustainable agriculture, biosynthetic genes

## Abstract

Soil-borne fungal pathogens such as *Sclerotium* spp., *Rhizoctonia* spp., and *Macrophomina* spp. pose significant threats to global agriculture, with soybean crops among the most severely affected due to damping-off disease. These pathogens cause substantial yield losses, making their management a critical concern. In this study, we investigated the potential of *Bacillus siamensis* BCL, a novel Neotropical strain, as an eco-friendly solution for managing *Sclerotium*, *Rhizoctonia*, and *Macrophomina* species. The strain exhibited strong antifungal activity, significantly inhibiting fungal growth in vitro, with the greatest suppression observed against *Macrophomina* spp., reaching up to 81%. In vivo assays further confirmed the biocontrol potential of *B. siamensis*. When applied at 10^6^ colony-forming units (CFU)/mL, the strain reduced disease symptoms and improved plant growth parameters—including root length, shoot biomass, and leaf number—compared to untreated, infected controls. The protective effect varied by pathogen, with the most significant recovery in root length observed against *Macrophomina* spp. (85%) and *Sclerotium* spp. (78%). In preventive treatments, fermentation extracts of the *B. siamensis* strain suppressed disease progression, although they did not promote seedling growth. A genomic analysis of *B. siamensis* BCL revealed genes encoding antimicrobial secondary metabolites, including terpenes, fengycins, and surfactins. These findings highlight *B. siamensis* BCL as a promising candidate for sustainable crop protection and a valuable resource for developing novel antimicrobial strategies in agriculture.

## 1. Introduction

*Bacillus siamensis* is an endophytic, spore-forming, Gram-positive bacterium belonging to the *Bacillus amyloliquefaciens* group [1,2]. Amid growing concerns about the environmental and health impacts of synthetic fungicides, *B. siamensis* has emerged as a promising biocontrol agent for managing soilborne pathogens [3,4]. This bacterium has shown potential in controlling pathogens that affect key crops such as soybean (*Glycine max*), tomato, sugarcane, banana, and wheat [5,6,7].

Phytopathogenic fungi, including *Fusarium* spp., *Sclerotium* spp., and *Macrophomina* spp., pose serious threats to agricultural productivity due to their ability to cause devastating diseases. These fungi employ diverse pathogenic mechanisms, such as toxin production and secretion of cell wall-degrading enzymes [8,9]. Additionally, they form resistant structures like spores and sclerotia, which enable persistence under harsh environmental conditions [10,11]. Some pathogens further weaken plant health by suppressing host immune defenses, thereby facilitating colonization and disease progression [12]. These multifaceted pathogenic strategies, combined with their adaptability to changing climates, complicate disease management—particularly under climate-induced stress.

In soybean crops, these pathogens cause diseases such as charcoal rot (*Macrophomina* spp.), southern blight (*Sclerotium* spp.), and damping-off and root rot (*Rhizoctonia* spp.), primarily affecting young plants. The severity of these diseases is strongly influenced by regional environmental conditions. Infections hinder seed germination, reduce plant vigor, and increase vulnerability to abiotic stress, further exacerbating agricultural challenges [13,14,15,16,17,18]. Yield losses can reach up to 60%, with severe outbreaks resulting in plant mortality rates of up to 90% during early developmental stages [4,19,20,21,22,23,24].

Currently, the management of soybean diseases relies heavily on chemical fungicides. However, their excessive use raises serious health and environmental concerns. Overuse has led to increased pathogen resistance and the contamination of ecosystems [5,25,26]. Additionally, fungicides disrupt soil microbial communities, reducing biodiversity and compromising long-term soil health and productivity [27].

*Bacillus* species are well known for producing antimicrobial compounds that inhibit fungal growth [28,29]. Among them, *B. siamensis* demonstrates significant biocontrol potential through the production of lipopeptides and antibiotics, including iturins, fengycins, and surfactins, as well as hydrolytic enzymes. These compounds disrupt fungal cell membranes and metabolic processes, thereby preventing spore germination and fungal proliferation [30,31]. Furthermore, *B. siamensis* can induce systemic resistance in plants by upregulating immune-related genes and colonizing root tissues during pathogen attacks, enhancing plant defense mechanisms and stress resilience [12,30,31].

Although the use of *Bacillus* species for biological control is well documented, further exploration is needed to fully understand their potential [32]. A promising avenue involves discovering novel *Bacillus* strains from underexplored habitats, which may exhibit broad-spectrum antagonism and plant growth-promoting traits. For example, isolates from ginger rhizosphere and tea garden soils have demonstrated antibacterial properties and growth enhancement effects [31,33,34]. Expanding this knowledge base may not only improve the applicability of *Bacillus* strains across diverse agricultural systems but also shed light on the largely underexplored interactions between plants and beneficial bacteria. These insights can support the development of more resilient and sustainable crop management strategies across various species [35,36].

Therefore, this study aims to (a) evaluate the in vivo and in vitro fungistatic effects of *Bacillus siamensis* against soilborne fungi (*Macrophomina* sp., *Rhizoctonia* sp., and *Sclerotium* sp.) associated with plant blight, (b) investigate the bacterium’s potential as a soybean growth promoter, and (c) identify genes involved in the biosynthesis of its antagonistic metabolites.

## 2. Materials and Methods

### 2.1. Soil-Borne Pathogens and Bacillus siamensis

The phytopathogenic fungi (*Sclerotium* sp., *Rhizoctonia* sp., and *Macrophomina* sp.) used in this study were obtained from the culture collection of the Molecular Biology Laboratory at the Federal University of Tocantins (Gurupi, Tocantins, Brazil). The fungi collection was built by isolating fungi from diseased soybean plants and confirmed as phytopathogenic through morphological analysis. The fungi were cultured on Potato Dextrose Agar (PDA) medium prepared with 4 g/L potato extract, 20 g/L dextrose, and 15 g/L agar, with the pH adjusted to 6.4 ± 0.2. Cultures were incubated at 28 ± 2 °C for 48 h under a 12-h photoperiod. The bacterial *B. siamensis* strain, previously isolated from soil samples of Gurupi county, was evaluated for its antagonistic activity against these fungi. The isolate was cultivated in Luria-Bertani (LB) medium composed of 5 g/L yeast extract, 10 g/L peptone, and 10 g/L NaCl. Cultures were incubated at 28 ± 2 °C under aerobic conditions. All fungal and bacterial cultures were stored under refrigeration until use.

Morphological analysis of bacterial isolates was performed using scanning electron microscopy (SEM). For this, an active colony was sampled and mounted on a silicon-coated slide, then processed for SEM imaging to observe morphological features.

### 2.2. Genomic Analysis and Sequencing of Bacillus siamensis

Genomic DNA was extracted using the Wizard^®^ Genomic DNA Purification Kit (Promega, Madison, WI, USA) according to the manufacturer’s instructions. Genome sequencing was conducted using Illumina MiSeq technology (TruSeq Nano DNA Kit, San Diego, CA, USA), with paired-end reads of 2 × 151 bp, an average insert size of 200 bp, and 100× coverage.

Quality analysis of the sequencing libraries was performed using FastQC v0.11.9, and low-quality reads were trimmed using Geneious v10.2.6 [37]. De novo genome assembly was conducted with SPAdes v3.10.0 using default parameters [38], and the final assembly quality was evaluated with QUAST v5.0.2 [39].

Genome annotation was performed using the NCBI Prokaryotic Genome Annotation Pipeline (PGAP). Functional genomic analysis was carried out with RASTtk 2.0 [40], focusing on identifying genes associated with secondary metabolite synthesis. Particular attention was given to genes involved in the biosynthesis of non-ribosomal peptides and polyketides, identified using antiSMASH (https://antismash.secondarymetabolites.org; acessed on 11 June 2023) [41]. A circular genome map was generated using the Proksee tool (https://proksee.ca; accessed on 19 December 2023) [42]. The genome sequence was deposited in GenBank under accession number NZ_JAWDKG000000000.

### 2.3. Phylogenetic Analysis and Average Nucleotide Identity of Bacillus siamensis

Sequence alignment and phylogenetic tree construction were performed using MEGA X [43]. A phylogenetic tree based on the *gyrB* gene (encoding DNA gyrase subunit B) was constructed, incorporating sequences of closely related *Bacillus* spp. retrieved from GenBank. The tree was built using the Neighbor-Joining (NJ) method.

Average Nucleotide Identity (ANI) analysis was conducted using JSpeciesWS [44], with the Tetra Correlation Search (TCS) function used to select related genomes. A heatmap dendrogram was generated using the Morpheus tool (https://software.broadinstitute.org/morpheus; accessed on 3 February 2024) to visualize relationships among strains.

### 2.4. In Vitro Antagonistic Activity of Bacillus siamensis Against Soil Pathogens

The antagonistic activity of *B. siamensis* against *Sclerotium* sp., *Rhizoctonia* sp., and *Macrophomina* sp. was evaluated using an in vitro assay. Seven-millimeter-diameter discs of fungal mycelium from actively growing cultures were placed at the center of Petri dishes containing PDA medium. *B. siamensis* cultures, adjusted to a concentration of 10^8^ colony-forming units (CFU)/mL, were inoculated 2.5 cm away from the center of each plate.

All plates were incubated in a biochemical oxygen demand (BOD) incubator at 28 ± 1 °C with a 12-h photoperiod for 7 days. Control plates contained only the fungal isolates, grown under identical conditions but without bacterial inoculation.

Fungal radial growth was measured periodically, and the percentage of growth inhibition was calculated using the following equation [45]:

$$I\;\%=\left(\frac{C-T}{C}\right)\times 100$$

where I % = percentage of mycelial growth inhibition; *C* = control radial growth (mm); and *T* = radial growth of treatment (mm). Treatments with no growth inhibition received a value of zero.

### 2.5. In Vivo Antagonistic Activity of Bacillus siamensis Against Soil Pathogens

The in vivo antagonistic effect of *B. siamensis* was evaluated using soybean cultivar 843680 (Agro Amazônia^®^, Cuiaba, MT, Brazil), following the methodology of Gilbert, et al. [46]. Soybean seeds were surface disinfected by immersion in 70% ethanol for 1 min, followed by 2.5% sodium hypochlorite for 4 min, and rinsed three times with sterile distilled water. The seeds were then air-dried.

Dried seeds were soaked in a bacterial suspension (10⁸ CFU/mL of *B. siamensis*) for 12 h. Four treated seeds were placed in a circular arrangement on a PDA-containing Petri dish, 2.5 cm away from the dish’s center. A 7 mm-diameter mycelial disc of the target phytopathogen (*Sclerotium* sp., *Rhizoctonia* sp., or *Macrophomina* sp.) was placed in the center.

Control plates were inoculated with fungal mycelium without the presence of *B. siamensis*. All treatments were conducted in biological triplicates, and all plates were incubated under the same conditions as the in vitro bioassays (28 ± 1 °C, 12-h photoperiod, 7 days). The percentage of mycelial growth inhibition was calculated using the same procedures applied in the in vitro bioassays.

### 2.6. Effect of Bacillus siamensis on Soybean Seed Germination and Growth

To evaluate the potential of *B. siamensis* in promoting soybean growth, a seed germination test was conducted using soybean cultivar 843680 (Agro Amazônia^®^, Brazil), following the RAS methodology [46]. Seeds were treated with a bacterial suspension containing 10^8^ CFU/mL of *B. siamensis*, using solutions ranging from 10% to 100% concentration.

Twenty treated seeds were placed 2 cm apart in Gerbox boxes lined with moistened absorbent paper. The boxes were incubated at 25 ± 1 °C under a 12-h photoperiod for 15 days, with daily moisture maintenance by spraying distilled water onto the paper.

The experiment included 20 replicates, and the following parameters were assessed at the end of the incubation period: shoot and root length, number of leaves and secondary roots, and biomass of both aerial and root parts. Biomass was determined by drying the samples in an oven at 65 °C until a constant weight was achieved.

### 2.7. In Vivo Antagonist Bioassay of Bacillus siamensis in Soybean

For the in vivo bioassay, treated seeds were sown on a layer of moistened clay plaster (0.5% phosphoric acid) covered with a 1 cm layer of sand. The experiment was conducted in a growth chamber for 21 days. At the cotyledonary vegetative stage [47], seedlings were inoculated with a suspension containing 10^8^ spores/mL of each pathogen. Two treatment strategies were evaluated: (1) a curative approach: the application of *B. siamensis* suspension 24 h after pathogen inoculation; (2) a preventive strategy: the application of *B. siamensis* suspension prior to pathogen inoculation.

Antagonistic efficacy was evaluated based on disease incidence and the degree of pathogen suppression. Morphological parameters, as outlined in the germination test, were recorded at the end of the experiment. Treatments followed a randomized complete block design with six replicates, including both positive and negative controls.

### 2.8. Statistical Analysis

All statistical analyses were performed using Sigma Plot 12.5 (Systat Software, San Jose, CA, USA) Data are expressed as mean ± standard deviation (SD). An unpaired Student’s *t*-test was used for pairwise comparisons, while one-way ANOVA (Kruskal–Wallis), followed by Tukey’s post hoc test, was applied for multiple group comparisons.

Figures were generated using SigmaPlot v12.5 and further edited for color and labeling in CorelDR W AGraphics Suite 2024.

## 3. Results

### 3.1. Identification of Soil-Borne Pathogens

**Figure 1:**
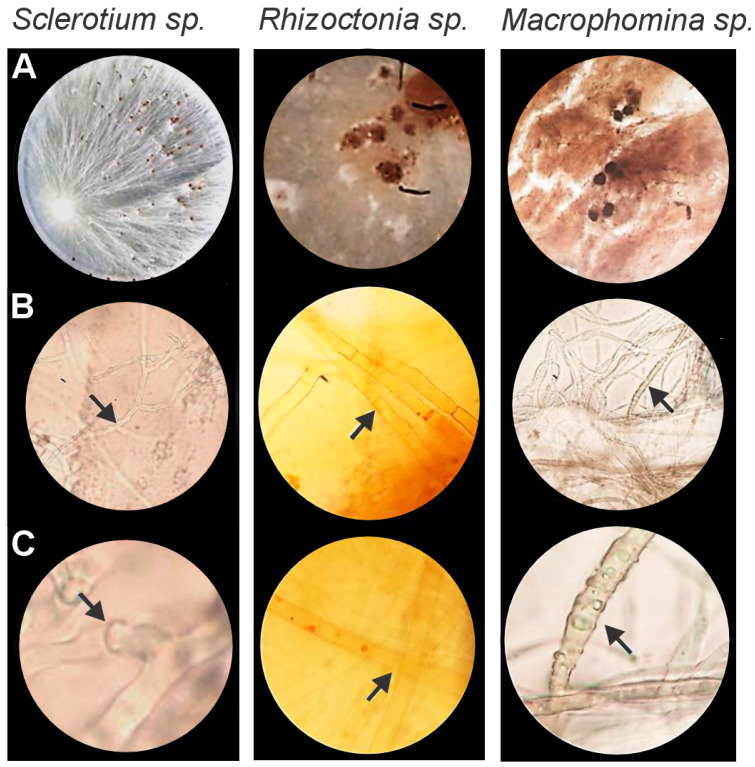
Morphological identification of phytopathogenic fungi: *Sclerotium* sp. (left panels), *Rhizoctonia* sp. (central panels), and *Macrophomina* sp. (right panels), grown on potato dextrose agar (PDA). (**A**) Mycelium and sclerotia development. (**B**) Septate hyphae with multinucleated cells. (**C**) Clamp connections highlighting species-specific hyphal features. Arrows indicate clamp connections or distinctive cellular structures such as sclerotia initials or chlamydospores.

Three potential pathogens were isolated from soybean plants and identified at the genus level based on morphological and microscopic characteristics. Distinct variations in colony color, mycelial growth, spore formation, and hyphal structure enabled classification as *Sclerotium* sp., *Rhizoctonia* sp., and *Macrophomina* sp. (Figure 1).

For *Sclerotium* sp., mycelial growth was completed within four days, forming colonies with a white, cotton-like texture. By day 7, the sclerotia had turned brownish. Microscopic examination revealed septate hyphae with multinucleated cells and characteristic staple-like connections.

*Rhizoctonia* sp. exhibited rapid growth, covering the entire Petri dish within two days (Figure 1). Initially, the mycelium appeared white, gradually developing a brownish, cottony texture with irregular, lump-like sclerotia by day 7. Septate hyphae with characteristic 90° branching angles were observed.

*Macrophomina* sp. initially produced white mycelium that gradually turned grayish-brown, eventually forming dark brown microsclerotia. Prominent colony growth was observed after six days, accompanied by the presence of aggregated hyphae (Figure 1).

### 3.2. Characterization of Bacillus siamensis

**Figure 2:**
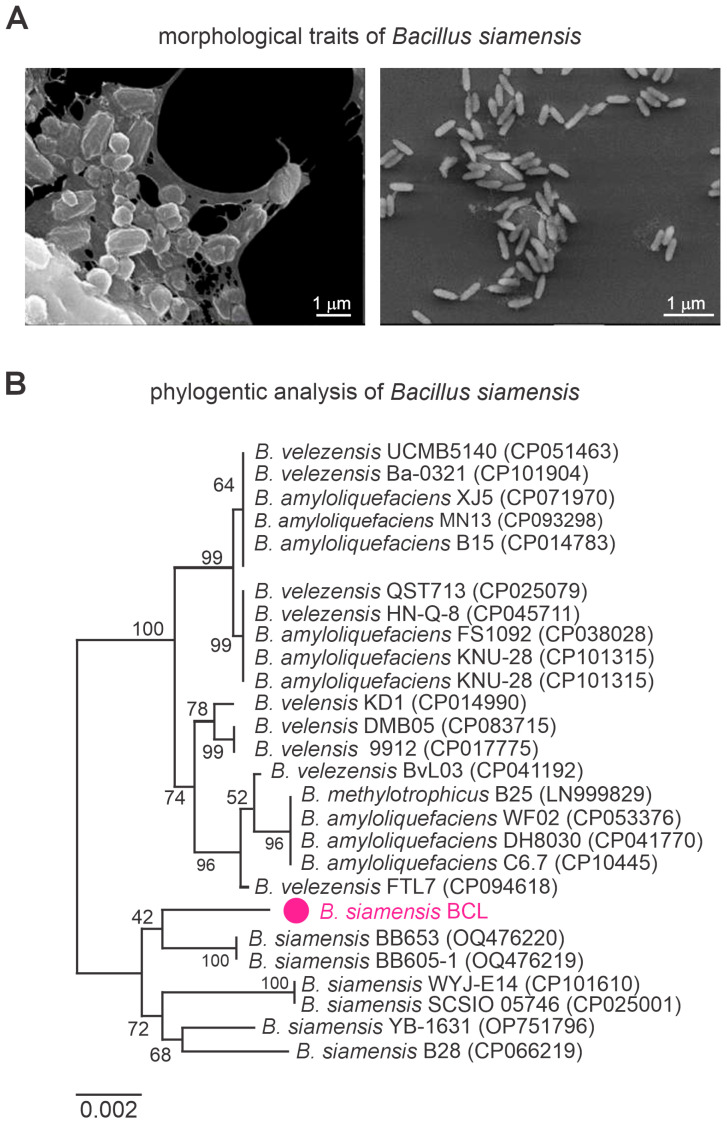
Scanning electron micrographs (SEM) of *Bacillus siamensis* (**A**) and phylogenetic tree analysis based on the *gyrB* gene (**B**), showing the taxonomic position of *B. siamensis* strain BCL within the *Bacillus* genus (highlighted in magenta). Average Nucleotide Identity (ANI) was calculated from whole-genome comparisons between *B. siamensis* BCL and 29 related strains. Numbers at the nodes indicate bootstrap support values based on 1000 replicates. The scale bar represents 0.0020 nucleotide substitutions per site.

Scanning Electron Microscopy (SEM) analysis of *B. siamensis* revealed its characteristic rod-shaped morphology, typical of the *Bacillus* genus. At 10,000× magnification, bacterial cells appeared in aggregated clusters, interconnected by visible extracellular material. At 1500× magnification, cells were more dispersed yet maintained a uniform, elongated shape. The bacterial surface was smooth and consistent across both magnifications, indicating a stable and homogeneous population (Figure 2A). These morphological traits confirm the identity of *B. siamensis* and support its potential for functional applications.

Phylogenetic analysis based on the 16S rDNA region (1351 bp) positioned the isolate within the *Bacillus* genus (Figure 2B). Genome-wide ANI analysis showed values exceeding 95% when compared with other *B. siamensis* strains, further confirming its classification within the species (Supplementary Figure S1).

### 3.3. Antagonistic Activity of Bacillus siamensis

**Figure 3:**
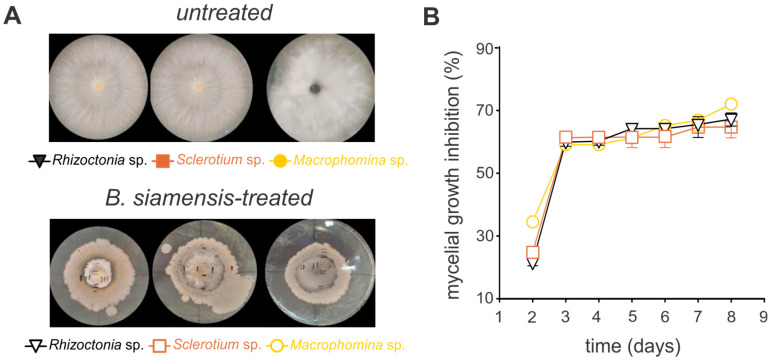
*Bacillus siamensis* BCL exhibits broad-spectrum antifungal activity against the phytopathogenic fungi *Sclerotium* sp., *Rhizoctonia* sp., and *Macrophomina* sp. grown on potato dextrose agar (PDA). (**A**) Illustrative images of the in vitro mycelial growth. “Untreated” represents the quantitative measurement of mycelial growth in the absence of *B. siamensis*, while “*B. siamensis*-treated” indicates fungal growth following the application of *B. siamensis*. (**B**) Quantitative inhibition of the in vitro mycelial growth. Each symbol represents the mean ± standard deviation of three replicates.

**Figure 4:**
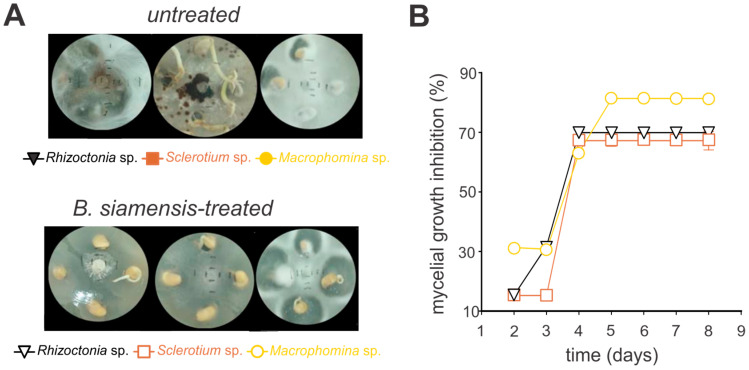
In vitro antifungal efficacy of crude extract from *Bacillus siamensis* against *Sclerotium* sp., *Rhizoctonia* sp., and *Macrophomina* sp. in soybean seeds. Seeds were incubated for seven days and allowed to germinate on potato dextrose agar (PDA). (**A**) Illustrative images of the in vitro mycelial growth. “Untreated” represents the quantitative measurement of mycelial growth in the absence of *B. siamensis*, while “*B. siamensis*-treated” indicates fungal growth following the application of *B. siamensis*. (**B**) Quantitative inhibition of the in vitro mycelial growth. Each symbol represents the mean ± standard deviation of three replicates.

The antifungal activity of *B. siamensis* was evaluated against the phytopathogenic fungi *Sclerotium* sp., *Rhizoctonia* sp., and *Macrophomina* sp. on agar plates, demonstrating significant efficacy against all tested pathogens (Figure 3 and Figure 4). For instance, the growth inhibition for all fungi species was markedly reduced, with over 50% inhibition observed by day three, stabilizing at approximately 70% thereafter (Figure 3, Supplementary Figure S2). Notably, in the in vitro bioassays using soybean seeds, *Macrophomina* sp. exhibited the highest level of inhibition, peaking at 81% (Figure 4, Supplementary Figure S3).

### 3.4. Effect of Bacillus siamensis on Soybean Growth and Protection

**Figure 5:**
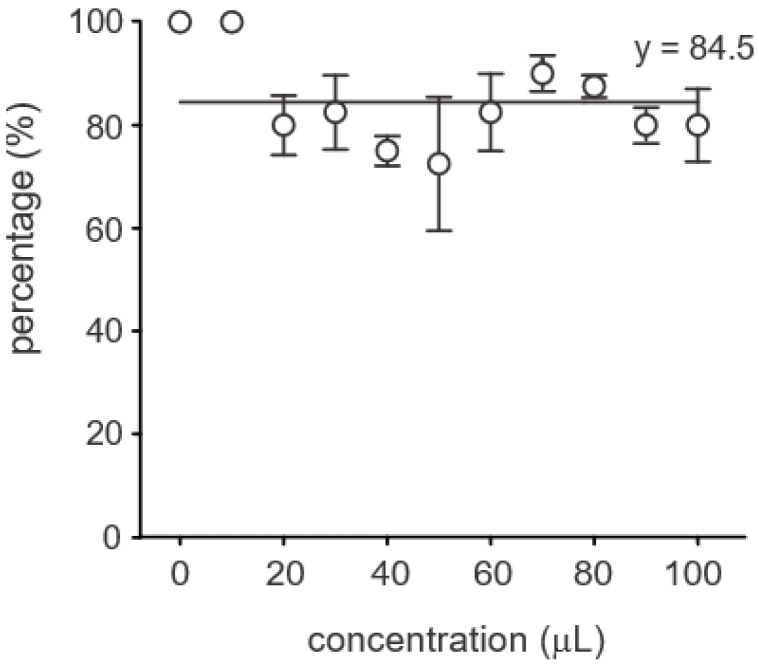
Germination rate of soybean seeds under different concentrations of *Bacillus siamensis* BCL. No significant differences were observed among treatments, indicating that bacterial concentration did not affect seed germination. Each point represents the mean (±standard deviation) of three replicates.

The application of *B. siamensis* supernatant to pathogen-challenged soybean plants elicited varied responses across several growth parameters, including seed germination, stem and root length, leaf number, secondary root formation, and biomass. Seed germination rates exceeded 60% across all treatments, with no significant differences observed between concentrations (Figure 5).

**Figure 6:**
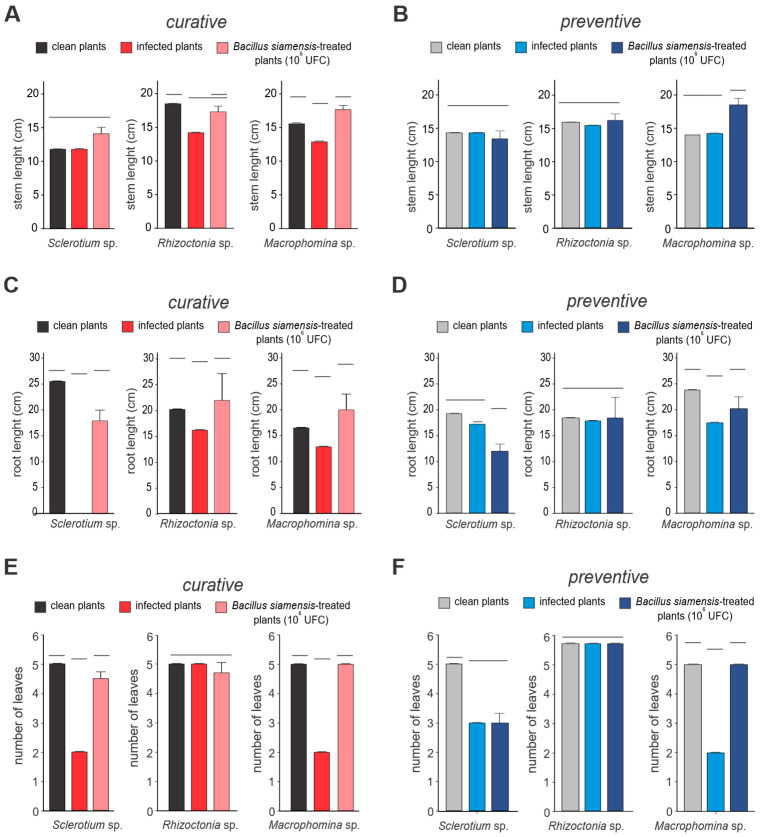
Growth promotion conferred by *Bacillus siamensis* BCL in soybean. Growth parameters—(**A**,**B**) stem emergence rate, (**C**,**D**) root length, and (**E**,**F**) number of leaves—were analyzed in the presence (preventive treatment) and absence (curative treatment) of *B. siamensis* against the fungal pathogens *Rhizoctonia* sp., *Sclerotium* sp., and *Macrophomina* sp. Curative treatment data are shown in red and pink, while preventive treatments are represented in shades of blue. Bars grouped at the same horizontal lines indicate the absence of significant differences according to Student’s *t*-test (*p* < 0.05). Histograms grouped by the same horizontal lines do not present statistical differences.

The application of *Bacillus* (10^8^ CFU/mL) significantly influenced plant growth parameters in soybean plants infected with the fungal pathogens *Rhizoctonia* sp., *Sclerotium* sp., and *Macrophomina* sp., with varying degrees of damage observed among the pathogens. When applied curatively after pathogen infection, *Bacillus* led to significant improvements in root length (40%), shoot biomass (35%), and leaf number (30%), all showing statistically significant differences (*p* < 0.05). The biocontrol activity of *Bacillus* was particularly effective against *Macrophomina* sp. and *Sclerotium* sp., with root length restoration reaching 85% and 78%, respectively (*p* < 0.01). In contrast, protection against *Rhizoctonia* sp. was less pronounced, with only 65% of root length loss restored (*p* < 0.05), suggesting that *Rhizoctonia* sp. exhibits greater resistance to *Bacillus* treatment. Although growth improvements were observed following *Bacillus* application after pathogen infection, recovery was less substantial compared to preventive treatment, underscoring the greater efficacy of *Bacillus* when applied prior to pathogen exposure (Figure 6). Treated shoots displayed improved initial stem and root growth compared to controls, regardless of the pathogen used.

Over time, roots from plants pre-treated with *B. siamensis* remained healthy, whereas those in pathogen-only treatments exhibited discoloration (dark brown or blackened roots), reduced secondary root development, and diminished leaf formation. These observations indicate that *B. siamensis* effectively prevented infection. Regarding biomass, shoots infected by *Rhizoctonia* sp. experienced a significant reduction in aerial biomass, while no significant changes were noted for other treatments. This highlights the potential of *B. siamensis* as a biocontrol agent in enhancing soybean resilience to fungal pathogens.

### 3.5. Genomic Insights into Secondary Metabolites Biosynthesis in Bacillus siamensis

**Figure 7:**
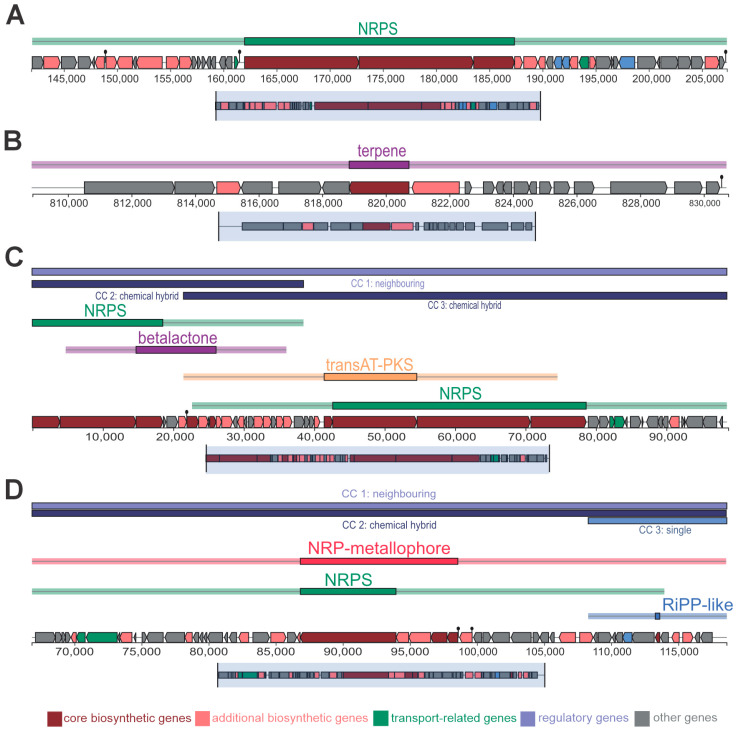
Secondary metabolite biosynthesis gene clusters. (**A**) Contig 1, showing (from top to bottom): Terpene 1; *Macrolactin H* (100% gene identity); *Bacillaene* (100% identity); *Fengycin* (100% gene identity); Terpene 2; and *Difficidin* (100% identity). (**B**) Contig 2, showing: *Bacillibactin* (100% identity) and *Bacilysin* (100% identity). Contig 3 showiing a *Lipopeptide* cluster (91% identity) in (**C**) and a hybrid NRPS/NRP-metallophore cluster spanning multiple catalytic genes and a RiPP-like gene cluster in (**D**). Genes are color-coded as follows: wine: core catalytic genes; salmon: accessory catalytic genes; blue: transport genes; and green: regulatory genes.

The genome assembly of *B. siamensis* yielded a total size of 3,853,558 bp distributed across 70 contigs, with a GC content of 46.05%. Annotation identified 3739 coding sequences (CDSs), including 14 rRNA and 77 tRNA genes (Supplementary Figure S4). Using the AntiSMASH tool, biosynthetic gene clusters (BGCs) were mapped throughout the genome (Figure 7, Supplementary Figure S4), highlighting the strain’s potential to produce bioactive compounds with biocontrol applications.

Contig 1 (Figure 7A) contained BGCs related to terpenes, non-ribosomal peptides (NRPS), and polyketides (PKS). These clusters indicate the strain’s ability to synthesize diverse antimicrobial and insecticidal compounds, including antibiotics and antifungals, which may be effective against plant pathogens and pests. Contig 2 (Figure 7B) identified two distinct clusters, one of which appears to encode pathways for non-ribosomal peptides. These pathways are known for their role in producing bioactive compounds with antifungal and antibacterial properties. Contig 3 presented another NRPS gene cluster, further emphasizing the strain’s potential for antimicrobial activity and NRP metallophore (Figure 7C,D).

These genomic insights confirm the functional capabilities of *B. siamensis* in biocontrol, driven by the production of a wide array of secondary metabolites, and underscore its utility in sustainable agricultural practices.

## 4. Discussion

Phytopathogenic fungi pose a significant threat to global agriculture, directly impacting economic stability and food security. The dynamics of plant–pathogen interactions are influenced by factors such as climate, geographical distribution, and cultivar susceptibility, all of which exacerbate infection processes [48]. Among soil-borne pathogens, genera such as *Macrophomina* sp., *Fusarium* sp., *Aspergillus* sp., *Pythium* sp., *Penicillium* sp., *Rhizoctonia* sp., *Verticillium* sp., and *Sclerotium* sp. are prominent contributors to crop diseases [49,50]. The morphological characterization of these pathogens provides crucial insights into their infection mechanisms and offers a foundation for the development of effective control strategies. These fungi infect both monocotyledonous and dicotyledonous plants, leading to diseases such as rot and wilt. They produce sclerotia-resilient survival structures that store reserve nutrients, allowing persistence under adverse environmental conditions [13,14,15,16,51]. The shape of sclerotia varies from rounded to irregular, aiding species identification [52]. *Rhizoctonia* species form colonies that transition from beige to brown and produce sclerotia with distinctive morphological features, which are key for classification [53]. Similarly, *Macrophomina* species are recognized for producing black, globose pycnidia visible on living plant tissues and other host structures [54,55]. The identification of these fungi, especially those targeting the root system, remains challenging. This complexity often delays timely control measures, leading to reduced crop productivity [56]. Moreover, increasing the resistance of phytopathogens to conventional fungicides underscores the urgent need for alternative control strategies. Exploring novel microbial isolates with biocontrol potential represents a promising avenue for sustainable disease management.

This study highlights the isolation and characterization of a novel strain of *B. siamensis* from soil samples collected in northern Brazil. Members of the *Bacillus* genus are widely recognized for their biocontrol capabilities due to their resilience to adverse environmental conditions, which allows them to thrive in diverse agricultural settings [57]. These bacteria produce a wide array of secondary metabolites, including lipopeptides and polyketides, which show selective toxicity against phytopathogens while being safe for non-target organisms [58,59]. These attributes make *B. siamensis* a promising candidate for use in organic farming and integrated pest management (IPM) systems [7,60].

Here, we demonstrated by in vitro assays the antagonistic activity of *B. siamensis* against pathogens such as isolates of *Sclerotium*, *Rhizoctonia*, and *Macrophomina* species that have shown their pathogenicity previously described [61,62,63,64]. Inhibition rates reached 59% early in the assessment, increasing to 70% by day seven, indicating sustained inhibitory activity. Preventing fungal spore germination is essential for maintaining plant and soil health, as it reduces the risk of pathogen dissemination in agricultural systems [31,57,65]. Such inhibition plays a critical role in managing soil-borne diseases like plant wilt, which significantly threaten crop productivity.

These findings align with previous research showing the efficacy of *B. siamensis* extracts in inhibiting mycelial growth and the sporulation of pathogenic fungi. For instance, *Bacillus* isolates have demonstrated complete inhibition of *Sclerotium rolfsii* [66], while *B. subtilis* and *B. subtilis spizizenii* effectively inhibited *Sclerotium* species [67]. In a study evaluating 22 rhizobacterial *Bacillus* isolates against *Macrophomina phaseolina*, 11 exhibited inhibitory activity, with *B. vallismortis* achieving 60% inhibition [68]. *B. amyloliquefaciens* has also shown strong antifungal activity against *Macrophomina* sp. [69]. Furthermore, previous investigations have shown that other *B. siamensis* strains controlled phytopathogen (e.g., *Fusarium* sp., *Alternaria* sp.) in crops such as banana, tomato, tobacco, and wheat [31,70,71,72]

In this study, the inhibitory effects of *B. siamensis* on soybean seeds infected with phytopathogenic fungi in PDA medium were particularly noteworthy. The isolate showed pronounced antifungal activity against *Macrophomina* sp., with inhibition reaching 81% by the fourth day and remaining stable throughout the cultivation period. Significant inhibition was also observed against *Rhizoctonia* and *Sclerotium*, both achieving rates of 70%. Compared to other studies, *B. siamensis* showed superior performance. For example, *Bacillus* species used in seed microbiolization in rice showed inhibition rates ranging from 19% to 60% [64].

In vivo experiments further assessed disease suppression in seedlings and revealed no significant differences in morphological variables between preventive and curative treatments with *B. siamensis*, indicating potential limitations under field conditions. The lack of observed efficacy may be due to the sterilization and composition of the sand used in pre-tests, which likely disrupted the rhizosphere microbiota and impaired microbial colonization. This finding supports previous studies that emphasize the role of soil microbiota and physicochemical properties in the effectiveness of *Bacillus* as a plant growth promoter [73,74,75].

Additionally, some phytopathogens produce plant hormones such as indoleacetic acid (IAA), which facilitate infection by enhancing host colonization [76,77]. Elevated levels of such hormones can disrupt plant development, further complicating the plant–pathogen interaction. These dynamics highlight the complexity of biological control, as pathogen-derived compounds may counteract the benefits of biocontrol agents. Here it is also crucial to recognize that the concentration and activity of antimicrobial compounds produced by *B. siamensis* are influenced by fermentation and cultivation conditions.

Environmental factors such as nutrient availability, temperature, and pH play critical roles in metabolite synthesis and, consequently, antifungal efficacy. Optimizing these parameters is essential to maximize *B. siamensis* performance in field applications. Previous studies have demonstrated that growth and metabolite production by *Bacillus* strains vary significantly with fermentation type and environmental conditions [77,78,79,80,81,82]. For instance, *B. subtilis* T9-05 produced higher levels of bacteriocins at neutral pH and moderate temperatures [81], and optimized cultivation conditions doubled bacteriocin yield in *Bacillus* species [82]. Similarly, enhanced production of antifungal compounds like iturin and fengycin was observed under controlled bioreactor conditions [16]. In this study, conventional culture methods and simple media may have limited metabolite production. Optimization is expected to increase yields and improve antifungal activity.

A genomic analysis of *B. siamensis* revealed significant biocontrol potential, including the ability to produce a broad range of bioactive compounds with pesticidal, antifungal, and antibacterial properties [83,84]. The presence of terpene, non-ribosomal peptide synthetase (NRPS), and polyketide synthase (PKS) clusters in contig 1 suggests the strain’s capacity to generate broad-spectrum antimicrobial compounds effective against fungi, bacteria, and viruses [6,51,85]. PKS-derived metabolites also enhance pest control mechanisms, further highlighting the strain’s agricultural potential. Gene clusters associated with surfactin production—a potent lipopeptide with antimicrobial and plant immunity-enhancing properties—were also identified. Surfactin has been shown to suppress pathogens such as *Botrytis cinerea* and *Rhizoctonia solani* [86,87]. These findings emphasize the versatility of *B. siamensis* as a promising biocontrol agent in sustainable agriculture.

The identification of hybrid clusters, notably NRP-metallophores in contig 2, is particularly noteworthy. Additionally, RiPP-like clusters suggest potential for bacteriocin synthesis, which plays a vital role in managing bacterial plant pathogens [88]. Other unexplored genomic regions point to novel biosynthetic pathways, indicating potential for discovering new bioactive compounds. This untapped genetic diversity emphasizes the biotechnological value of this *B. siamensis* strain for developing innovative agrochemicals and crop protection solutions.

Our findings demonstrate that *B. siamensis* has strong potential as a biocontrol agent against soil-borne pathogens. Its ability to produce a diverse arsenal of bioactive compounds, including antimicrobial peptides, siderophores, and hybrid metabolites, positions it as a valuable tool for integrated pest and disease management. This study provides a foundation for further research into its practical applications in agriculture, and future field trials will be essential to validate its efficacy under real-world conditions.

## 5. Conclusions

This study underscores the promising potential of *Bacillus siamensis* as a biocontrol agent against phytopathogenic fungi, particularly *Sclerotium* sp. In vitro assays demonstrated the strong inhibitory effect of the BCL isolate on pathogen growth, achieving up to 81% inhibition against *Macrophomina* sp. Genomic analysis further revealed gene clusters associated with the production of bioactive compounds such as terpenes, fengycins, and surfactin, indicating robust mechanisms for pathogen suppression. These findings align with previous research highlighting the antagonistic capabilities of *Bacillus* species and affirm the utility of *B. siamensis* in biological control strategies for plant diseases.

However, the study also highlights challenges associated with in vivo applications. The growth-promoting potential and antagonistic activity of *B. siamensis* were shown to be influenced by environmental factors, including the rhizosphere microbiota and external conditions such as pH, temperature, and nutrient availability. Optimizing these factors is essential to enhance the isolate’s efficacy under field conditions.

In conclusion, this study makes a significant contribution to the advancement of sustainable biotechnological solutions for integrated disease management and organic farming. The results position *B. siamensis* as a viable alternative to chemical pesticides, supporting ecological and sustainable agricultural practices. Future research should focus on field trials to validate its effectiveness under diverse environmental conditions and explore its integration into sustainable crop management systems.

## Data Availability

The 16 S rDNA sequence of *Bacillus siamensis* BCL was submitted to NCBI, and the accession ID was NZ_JAWDKG000000000.

## Supplementary Materials

The following supporting information can be downloaded at https://www.mdpi.com/article/10.3390/microorganisms13061366/s1, Figure S1: Average nucleotide identity (ANI) matrix comparing *Bacillus siamensis* BCL strain with other *Bacillus* species. The analysis was performed using the FastANI tool, with ANI values presented as percentages. Colors represent the degree of identity, ranging from green (high similarity, ≥97%) to pink/purple (low similarity, <90%). *Bacillus siamensis* BCL shows high similarity with other *B. siamensis* strains, confirming its taxonomic classification and distinguishing it from other species of the *Bacillus amyloliquefaciens* complex; Figure S2: *Bacillus siamensis* BCL exhibits broad-spectrum antifungal activity against the phytopathogenic fungi *Sclerotium* sp., *Rhizoctonia* sp., and *Macrophomina* sp. grown on potato dextrose agar (PDA). “Untreated” represents the quantitative measurement of mycelial growth in the absence of *B. siamensis*, while “*B. siamensis*-treated” indicates fungal growth following the application of *B. siamensis*. Each symbol represents the mean ± standard deviation of three replicates; Figure S3: In vitro antifungal efficacy of crude extract from *Bacillus siamensis* against *Sclerotium* sp., *Rhizoctonia* sp., and *Macrophomina* sp. in soybean seeds. Seeds were incubated for seven days and allowed to germinate on potato dextrose agar (PDA). “Untreated” refers to the quantitative measurement of mycelial growth in the absence of treatment, while “treated” indicates the percentage inhibition of mycelial growth following application of the crude extract. Each symbol represents the mean ± standard deviation of three replicates.; Figure S4: Genomic characterization of *Bacillus siamensis* BCL strain. (A) Circular map of the annotated *B. siamensis* BCL genome, from the outside to the inside showing: ring 1 for protein-coding sequences (CDS) in clockwise (purple) and counterclockwise (purple) directions, ring 2 for biosynthetic clusters of secondary metabolites identified by antiSMASH (red), ring 3 for RNA-coding regions (blue), ring 4 for GC skew (green/purple), and ring 5 for GC content (black). (B) Functional distribution of annotated genes, classified into 27 categories based on RAST subsystems, with a focus on amino acid metabolism, carbohydrate metabolism, and metabolism of cofactors, vitamins, and pigments. Genes related to stress resistance, sporulation, motility, iron acquisition, defense, and secondary metabolite production are also highlighted; Table S1: Putative gene clusters for the synthesis of secondary metabolites in the Bacillus siamensis BCL genome, identified by antiSMASH. The table lists the cluster types, their genomic locations (from and to), the most similar known cluster, and the similarity percentage. Identified clusters include genes involved in the biosynthesis of antimicrobial and bioactive compounds, such as fengycins, surfactins, bacillibactin, terpenes, and various NRPS and PKS types.
