# Supplementary material for: A Novel Neotropical Bacillus siamensis Strain Inhibits Soil-Borne Plant Pathogens and Promotes Soybean Growth
Source: Microorganisms. 2025 Jun 12;13(6):1366. doi: 10.3390/microorganisms13061366 (PMC12195227; doi:10.3390/microorganisms13061366)
Supplement: Supplementary file 1 [file microorganisms-13-01366-s001.zip › microorganisms-3690550-supplementary.pdf]

Supplementary Materials

A Novel Neotropical *Bacillus siamensis* Strain Inhibits Soil-Borne Plant Pathogens and Promotes Soybean Growth

Rodrigo F. Moreira [1,†], Elizabeth B. E. Pires [1,†], Odaiza F. Sousa [1], Giselly B. Alves [2], Luis O. Viteri Jumbo [1,3], Gil R. Santos [1], Luís J. Maia [1,4], Bergmann M. Ribeiro [4], Guy Smagghe [1,5,6,7], Elvio H. B. Perino [8], Rudolf Hausmann [8], Eugenio E. Oliveira [1,9,*] and Raimundo W. S. Aguiar [1,*]

[1] Programa de Pós-graduação em Biotecnologia, Universidade Federal do Tocantins (UFT), Gurupi 77402-970, TO, Brazil
[2] Departamento de Biotecnologia, Universidade Federal do Tocantins, Gurupi 77410-530, TO, Brazil; gybiotec@gmail.com
[3] Programa de Pós-graduação em Produção Vegetal, Universidade Federal de Tocantins (UFT), Gurupi 77402-970, TO, Brazil
[4] Departamento de Biologia Celular, Instituto de Biologia, Universidade de Brasília (UnB), Brasília 70910-900, DF, Brazil
[5] Institute of Entomology, Guizhou University, Guiyang 550025, China
[6] Department of Plants and Crops, Ghent University, 9000 Ghent, Belgium
[7] Department of Biology, Vrije Universiteit Brussel (VUB), 1050 Brussels, Belgium
[8] Department of Bioprocess Engineering, Institute of Food Science and Biotechnology, University of Hohenheim, Fruwirthstr. 12, 70599 Stuttgart, Germany
[9] Departamento de Entomologia, Universidade Federal de Viçosa, Viçosa 36570-900, MG, Brazil
* Correspondence: eugenio@ufv.br (E.E.O.); rwsa@uft.edu.br (R.W.S.A.)
† These authors contributed equally for the manuscript.

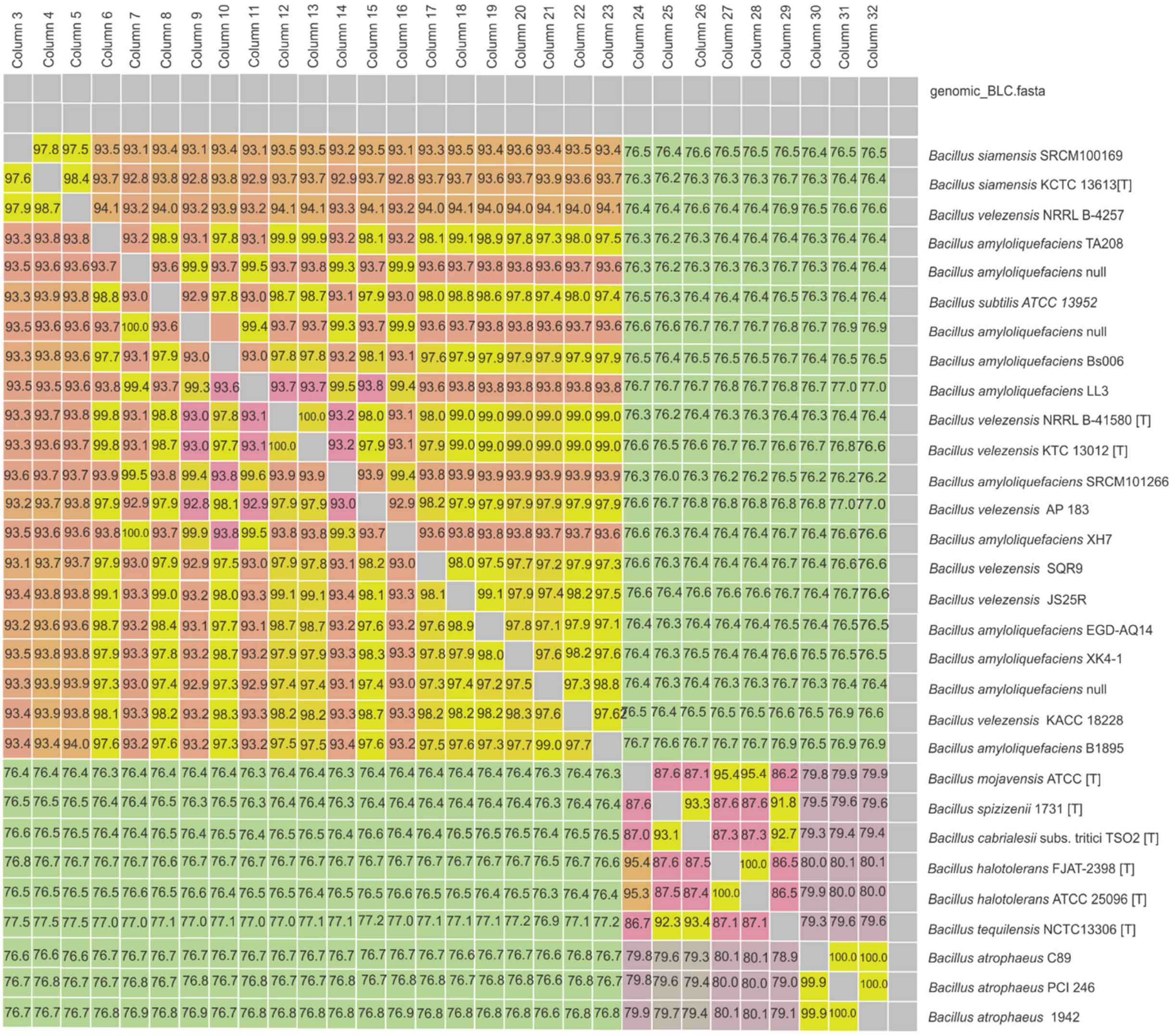

Supplementary Figure S1. Average nucleotide identity (ANI) matrix comparing *Bacillus siamensis* BCL strain with other *Bacillus* species. The analysis was performed using the FastANI tool, with ANI values presented as percentages. Colors represent the degree of identity, ranging from green (high similarity, ≥ 97%) to pink/purple (low similarity, < 90%). *Bacillus siamensis* BCL shows high similarity with other *B. siamensis* strains, confirming its taxonomic classification and distinguishing it from other species of the *Bacillus amyloliquefaciens* complex.

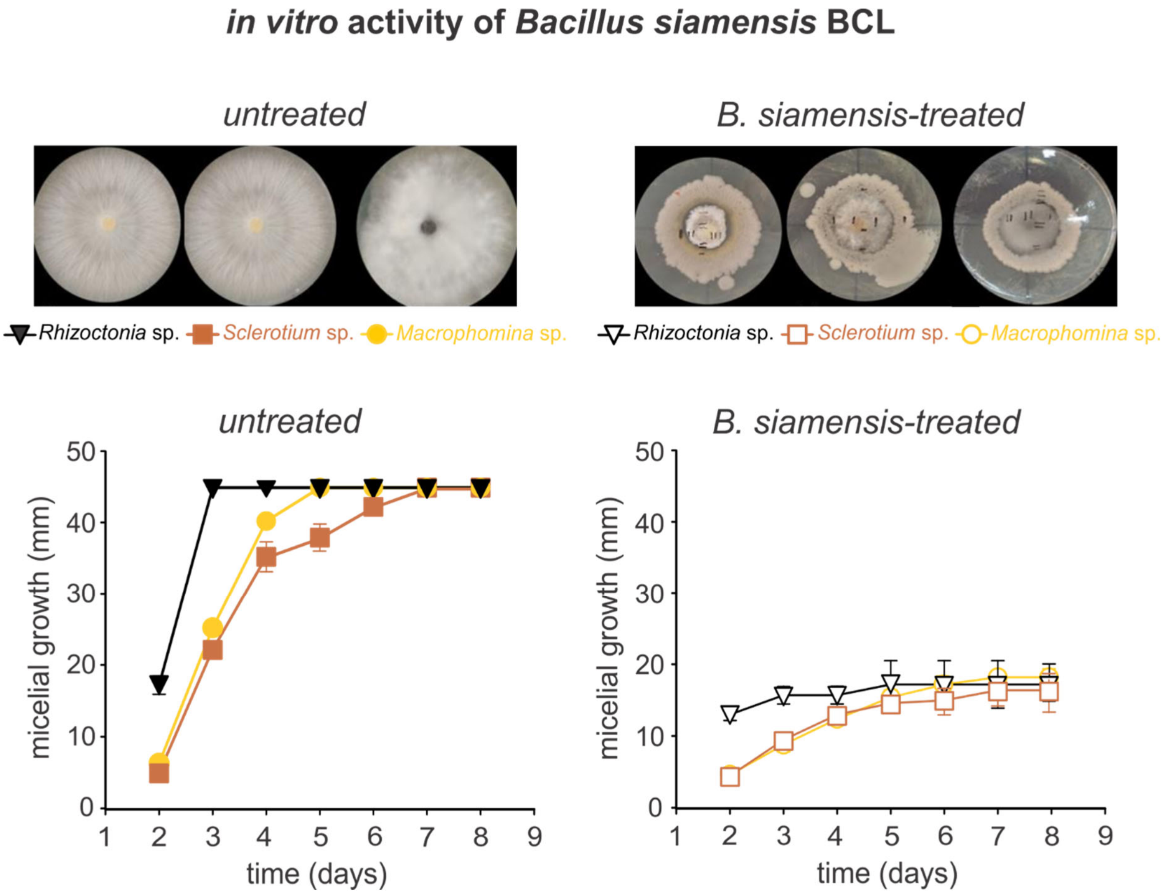

Supplementary Figure S2. *Bacillus siamensis* BCL exhibits broad-spectrum antifungal activity against the phytopathogenic fungi *Sclerotium* sp., *Rhizoctonia* sp., and *Macrophomina* sp. grown on potato dextrose agar (PDA). "Untreated" represents the quantitative measurement of mycelial growth in the absence of *B. siamensis*, while "*B. siamensis*-treated" indicates fungal growth following the application of *B. siamensis*. Each symbol represents the mean ± standard deviation of three replicates.

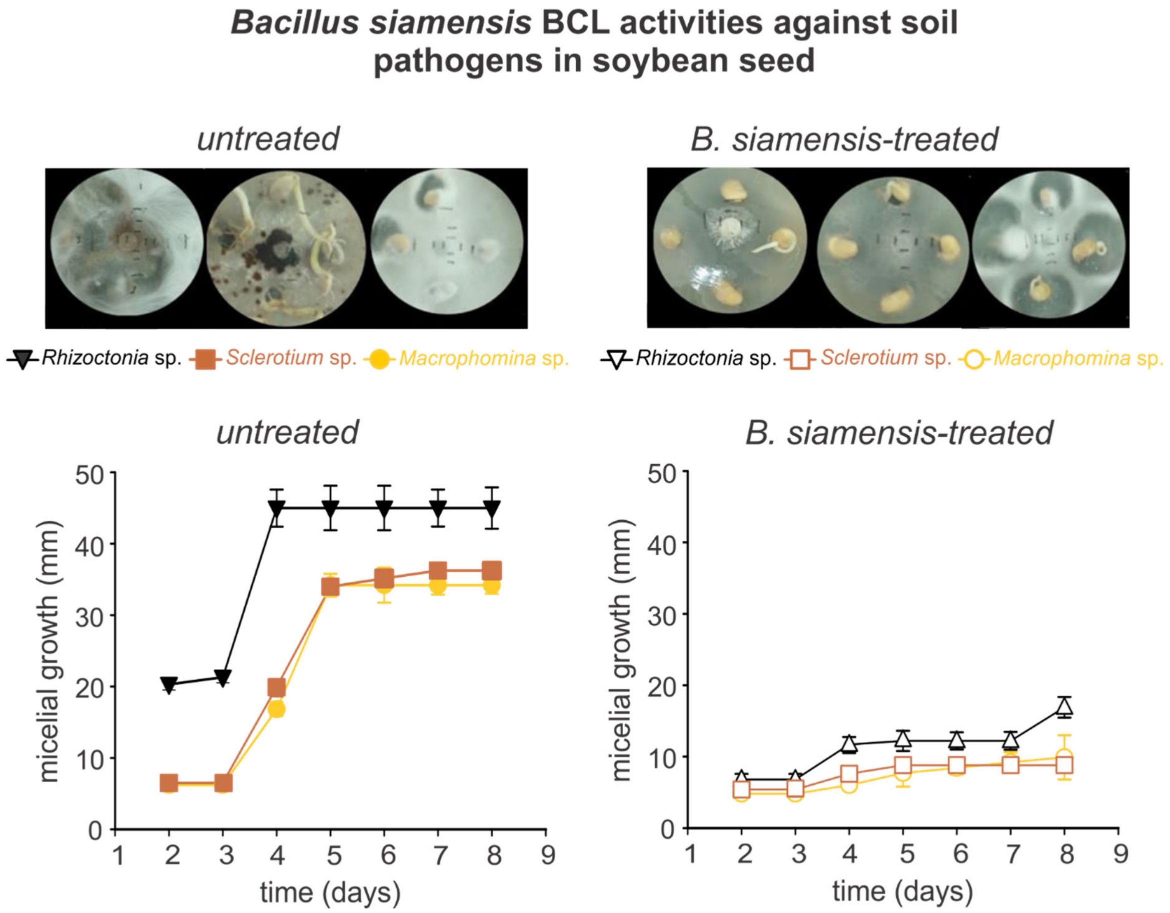

Supplementary Figure S3. *In vitro* antifungal efficacy of crude extract from *Bacillus siamensis* against *Sclerotium* sp., *Rhizoctonia* sp., and *Macrophomina* sp. in soybean seeds. Seeds were incubated for seven days and allowed to germinate on potato dextrose agar (PDA). "Untreated" refers to the quantitative measurement of mycelial growth in the absence of treatment, while "treated" indicates the percentage inhibition of mycelial growth following application of the crude extract. Each symbol represents the mean ± standard deviation of three replicates.

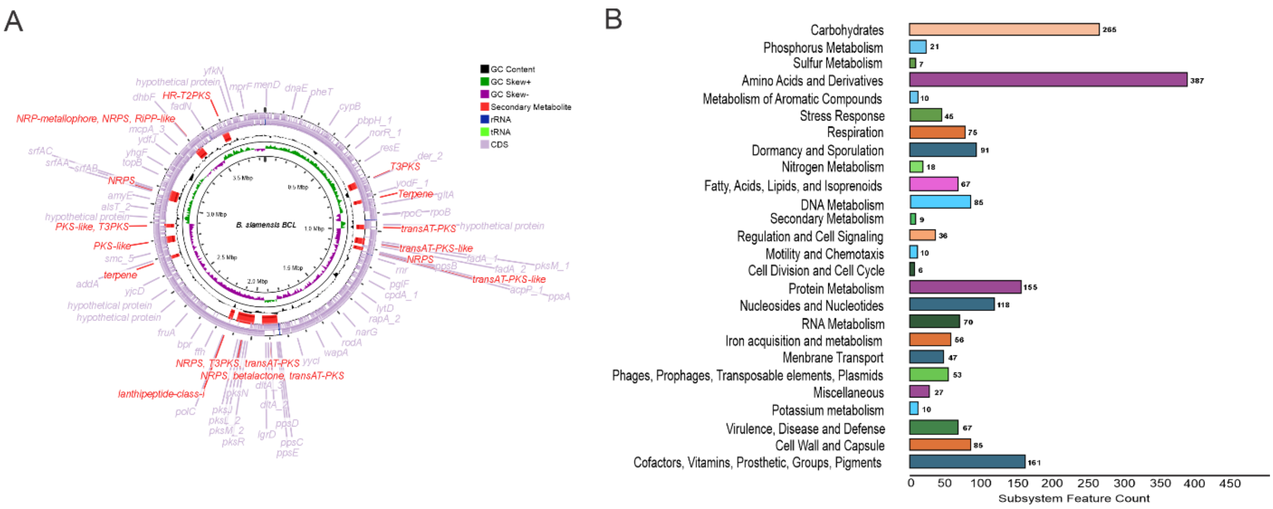

Supplementary Figure S4. Genomic characterization of *Bacillus siamensis* BCL strain. (***A***) Circular map of the annotated *B. siamensis* BCL genome, from the outside to the inside showing: ring 1 for protein-coding sequences (CDS) in clockwise (purple) and counterclockwise (purple) directions, ring 2 for biosynthetic clusters of secondary metabolites identified by antiSMASH (red), ring 3 for RNA-coding regions (blue), ring 4 for GC skew (green/purple), and ring 5 for GC content (black). (***B***) Functional distribution of annotated genes, classified into 27 categories based on RAST subsystems, with a focus on amino acid metabolism, carbohydrate metabolism, and metabolism of cofactors, vitamins, and pigments. Genes related to stress resistance, sporulation, motility, iron acquisition, defense, and secondary metabolite production are also highlighted.

Supplementary Table S1. Putative gene clusters for the synthesis of secondary metabolites in the Bacillus siamensis BCL genome, identified by antiSMASH. The table lists the cluster types, their genomic locations (from and to), the most similar known cluster, and the similarity percentage. Identified clusters include genes involved in the biosynthesis of antimicrobial and bioactive compounds, such as fengycins, surfactins, bacillibactin, terpenes, and various NRPS and PKS types.

Regions	Types	From	To	Most Similar Known Cluster	Similarity
1.1	T3PKS	692,721	733,821	-	-
1.2	Terpene	808,884	830,767	-	-
3.1	transAT-PKS	27,978-	57,385	Aurantinin B,C,D	35%
5.1	transAT-PKS-like	1	37,085	Aurantinin B,C,D	11%
7.1	NRPS	1	13,176	Plipastatin	23%
8.1	transAT-PKS-like	1	8,475	Aurantinin B,C,D	10%
23.1	NRPS, betalactone, transAT-PKS	1	98,765	Fengycin	86%
23.2	transAT-PKS, NRPS, T3PKS	147,583	257,624	Bacillaene	100%
23.3	lanthipeptide-class-i	283,788	310,104	-	-
34.1	Terpene	193,526	214,269	-	-
34.2	PKS-like	295,953	337,197	Butirosin A,B	7%
34.3	T3PKS, PKS-like	391,640	415,464	Aurantinin B,C,D	42%
45.1	NRPS	141,929	207,336	Surfactin	86%
56.1	NRP-metallophore, NRP, RiPP-like	66,809	118,593	Bacillibactin	100%
66.1	HR-T2PKS	69,163	111,331	-	-
